# Principles of human movement augmentation and the challenges in making it a reality

**DOI:** 10.1038/s41467-022-28725-7

**Published:** 2022-03-15

**Authors:** Jonathan Eden, Mario Bräcklein, Jaime Ibáñez, Deren Yusuf Barsakcioglu, Giovanni Di Pino, Dario Farina, Etienne Burdet, Carsten Mehring

**Affiliations:** 1 Department of Bioengineering, Imperial College of Science, Technology and Medicine, London, UK; 2 BSICoS, IIS Aragón, Universidad de Zaragoza, Zaragoza, Spain; 3 Department of Clinical and Movement Neurosciences, Institute of Neurology, University College London, London, UK; 4 NEXT: Neurophysiology and Neuroengineering of Human-Technology Interaction Research Unit, Università Campus Bio-Medico di Roma, Rome, Italy; 5 Bernstein Center Freiburg, University of Freiburg, Freiburg im Breisgau, 79104 Germany; 6 Faculty of Biology, University of Freiburg, Freiburg im Breisgau, 79104 Germany

**Keywords:** Biomedical engineering, Mechanical engineering

## Abstract

Augmenting the body with artificial limbs controlled concurrently to one’s natural limbs has long appeared in science fiction, but recent technological and neuroscientific advances have begun to make this possible. By allowing individuals to achieve otherwise impossible actions, movement augmentation could revolutionize medical and industrial applications and profoundly change the way humans interact with the environment. Here, we construct a movement augmentation taxonomy through what is augmented and how it is achieved. With this framework, we analyze augmentation that extends the number of degrees-of-freedom, discuss critical features of effective augmentation such as physiological control signals, sensory feedback and learning as well as application scenarios, and propose a vision for the field.

## Abstract (web summary)

In this Review, the authors discuss recent technological and neuroscientific advances in human body augmentation. They construct a movement augmentation taxonomy, discuss how it is achieved, and propose a vision for the field.

## Introduction

**Fig. 1:**
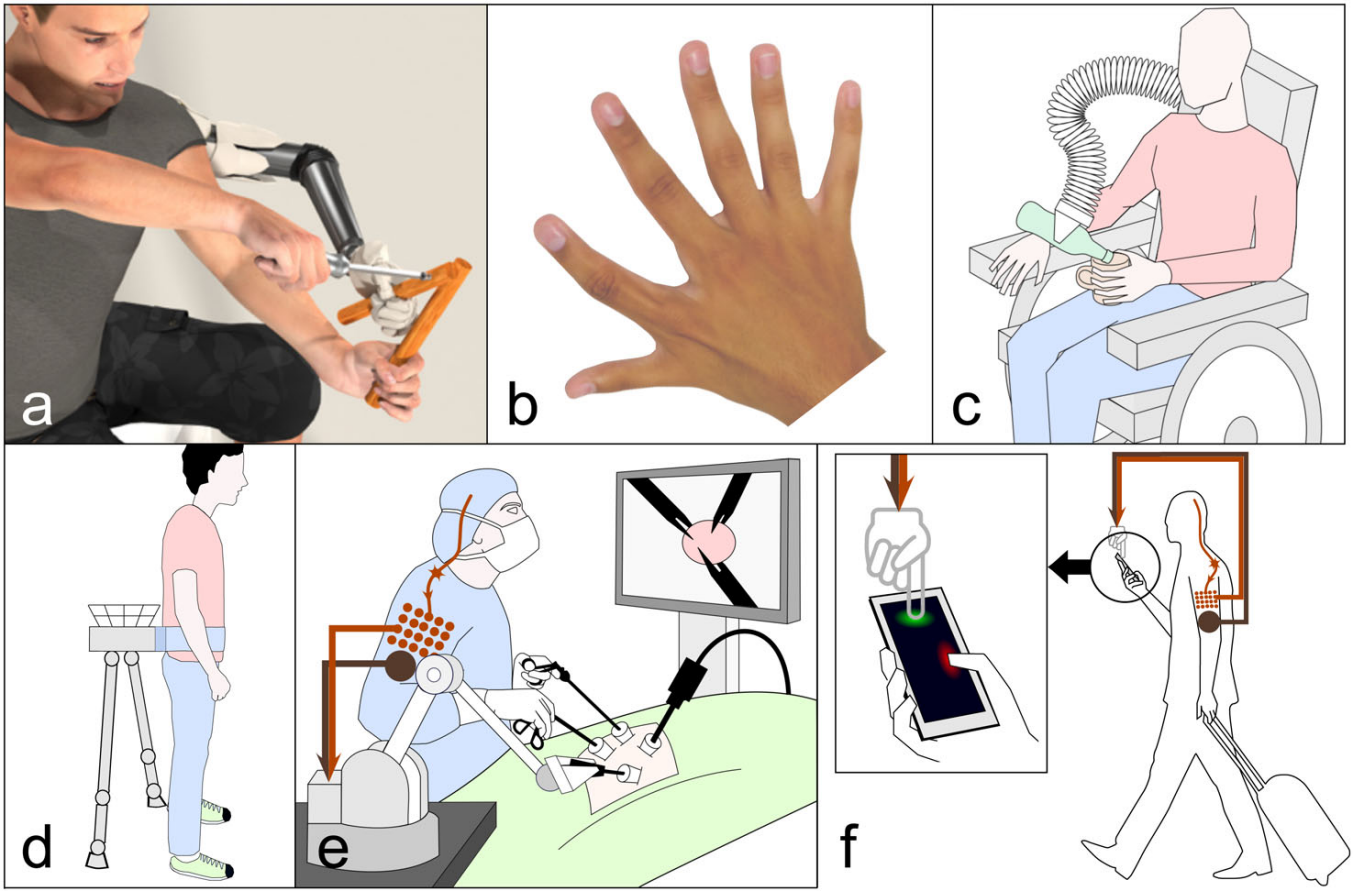
DoF augmentation concepts and natural augmentation. **a** Extra hand for assembly tasks. **b** Polydactyly hand with six fingers providing superior manipulation abilities^4^. **c** Third arm to facilitate activities of daily living in hemiplegics. **d** Centaur robot for stability and walking assistance. **e** Surgery with three tools controlled by the hands and a neural interface. **f** Augmented interaction with a mobile device can free one hand to e.g. operate a map. Panel **a** by Tobias Pistohl; **b** modified from ref. ^4^. Panels **c**, **e**, and **f** by Nathanael Jarrassé. Panel **d** by Camille Blondin.

The goal of *human movement augmentation* is to extend a person’s movement abilities. When this augmentation increases the number of movement degrees-of-freedom (*DoF augmentation*), it can enable a person to perform tasks that are impossible to achieve with their natural limbs alone. An example would be the third arm that a person can control simultaneously to their natural arms in trimanual tasks (Fig. 1a). In this emerging paradigm, a user is endowed with a *supernumerary effector* (SE) in the form of a wearable limb (Fig. 1a), an external robot (Fig. 1c, e), or an effector in virtual reality (Fig. 1f). While human movement augmentation is often considered for unimpaired individuals, it uses technologies that originate from developments to restore functions in impaired individuals, such as prosthetics for amputees or exoskeletons for stroke patients. However, it is free from the typical constraints of restoration neurotechnology: it does not need to substitute a lost ability with the same function, and it is not bound to a natural appearance. Such freedom to implement out-of-the-box solutions could in turn be used to apply SEs for rehabilitation/restoration or to develop new technologies for aiding impaired individuals (Fig. 1c, d).

Despite the recent growth in interest in DoF augmentation^1–3^, the realization of SEs that can be controlled independently from the natural limbs and in coordination with them has remained elusive. A fundamental open question is whether human users can control additional DoFs without limiting natural movement. In this regard, a recent study demonstrated that subjects born with an extra finger on each hand can control multiple extra DoFs, giving them superior manipulation abilities without any apparent movement deficits (Fig. 1b and ref. ^4^). However, it is unclear whether subjects can learn to control artificial *supernumerary DoFs* (sDoFs) that they are not born with, and whether this could enhance functional abilities. If so, where would such augmentation capabilities come from and what are their limits? How can the nervous system represent the extra limb and its relation to other limbs? These questions will impact the future of movement augmentation and determine which approaches are most suited.

This paper analyzes the potential and constraints for different DoF augmentation strategies by considering these questions. Compared to recent supernumerary robotic limb reviews^1–3^, particular emphasis is placed on the neuroscientific and technical factors that can enable the control of sDoFs, rather than on the specific device design and fabrication. In the first section we develop a taxonomy of movement augmentation, which yields the first classification of different types of augmentation. The components needed for augmentation are identified in the subsequent section. We then review and analyze the current implementations of DoF augmentation based on the proposed taxonomy, considering the potential features for each augmentation type. After we examine how the critical factors of feedback and learning affect these different augmentation types. Finally, we investigate the applications scenarios of DoF augmentation, and identify the impediments and open issues to make DoF augmentation a reality.

In this manuscript, relevant literature was identified using the following steps: (i) papers on supernumerary robotic limbs were first identified using the keywords “supernumerary robot” and “wearable robotic arm” as search terms in the IEEE explore, Web of Science, and Google Scholar search databases, over a period from 2000 to 2021. (ii) The 1340 papers found in this way were filtered to remove duplicate works and to ensure that the selected papers had a scope encompassing “experiments considering movement augmentation” or “the development or validation of a human augmentation control scheme”. This resulted in 95 manuscripts. (iii) Papers describing works on the same topic with similar device and/or applications were identified, and representative papers are discussed in the present manuscript. (iv) This review was complemented by author-specific knowledge with regards to the fields of “brain–computer interfaces”, “artificial proprioception” and “motor learning” which were included due to their relevance to topics discussed in later sections.

## A taxonomy of movement augmentation

**Fig. 2:**
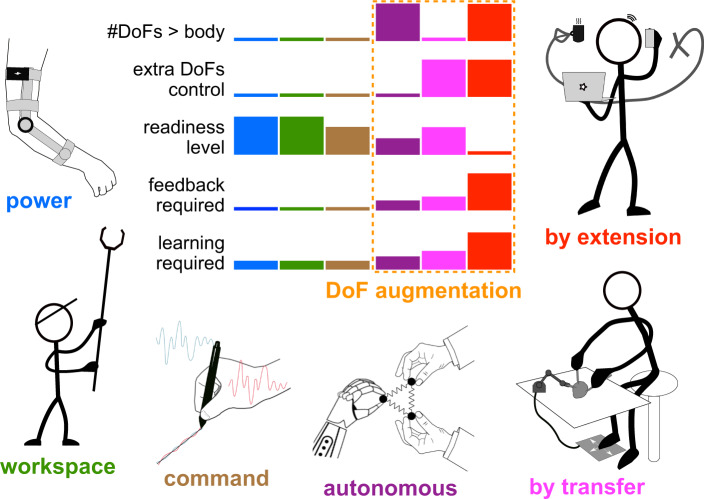
Schematic overview of the different concepts of human movement augmentation and qualitative analysis of major characteristics. The logos for each form of augmentation (figures by Camille Blondin) illustrate example applications. The five histograms correspond to whether or not the form of augmentation grants the user more DoFs than their body and provides them with explicit control, as well as the level of how ready each device is for widespread usage, and how much feedback and learning would be required.

Movement augmentation can be classified based on the specific aspects of motor action that are enhanced (Fig. 2). Existing forms of augmentation include:

- *Power augmentation* which increases the user’s forces or speed. Examples are exoskeletons^5^ and suits^6^ reducing physical load^7^ as well as cars increasing speed.
- *Workspace augmentation* which extends the spatial reach of natural limbs, with tools such as a rake or an endoscope^8^, or through teleoperation or outer space manipulation^9^.
- *Command augmentation* which addresses the limitations of the motor system by processing the user’s command signal. Examples include switching control between different tools in robot-aided surgery using a dedicated handle with a clutch^10^, or tremor attenuation to improve eye surgery through active noise cancellation with a robotic interface^11^.

These augmentation forms improve already existing movement abilities. In contrast, DoF augmentation endows subjects with extra abilities to interact with their environment. While it has only emerged in the last decades^12–14^, DoF augmentation could potentially reshape human-environment interaction as the examples of Fig. 1 illustrate.

DoF augmentation ideally provides independent and coordinated control of sDoFs with respect to one’s own natural DoFs. Hence, a mere increase in the number of mechanical DoFs is not sufficient for DoF augmentation as the additional DoFs also need to be controlled at least to some degree independently from the natural DoFs. This can be realized in three different ways (Fig. 2):

- *Autonomous DoF augmentation* extends the number of DoFs involved in one or more tasks using autonomously controlled devices. For instance, a robot may help carry an object with its human user.
- *DoF augmentation by transfer*, in contrast, lets the user control the sDoFs. However, it only extends the number of movement DoFs involved in a task by re-purposing other existing body DoFs that are task-irrelevant. An example would be a third arm controlled by foot movements for three-tool surgery.
- *Augmentation by DoF extension* lets the user control the sDoFs by extending the body’s total number of movement DoFs. An example would be a third arm driven by neural activity that can be controlled independently from and concurrently with the natural limbs while preserving the full repertoire of natural movement abilities.

The different ways to achieve DoF augmentation are analyzed in Fig. 2. While all forms of DoF augmentation may provide sDoFs, only augmentation by extension grants the user both an increased number of movement DoFs and their direct control. The figure also illustrates the differences between these augmentation schemes in terms of their readiness to be used, their potential requirement for additional feedback devices, and the learning required for their use.

The assumption of both augmentation by transfer and extension is that the human user is able to voluntarily manipulate body signals that do not interfere with natural motion behaviors. Limbs not involved in a task, such as the foot while seated in bimanual manipulation, could be used to enlarge the range of possible actions, enabling augmentation by transfer. In addition, as the number of muscles is higher than the body’s mechanical DoFs, there is muscle redundancy that could potentially be used for augmentation by transfer and extension. Moreover, the number of neurons used for musculoskeletal control is much higher than the number of muscles, suggesting further potential DoF augmentation capability. Such areas in the space of possible signals which do not correlate with differences in movements, have been coined a “null space”^15–17^ in analogy to the null space of linear algebra.

## Features of movement augmentation

**Fig. 3:**
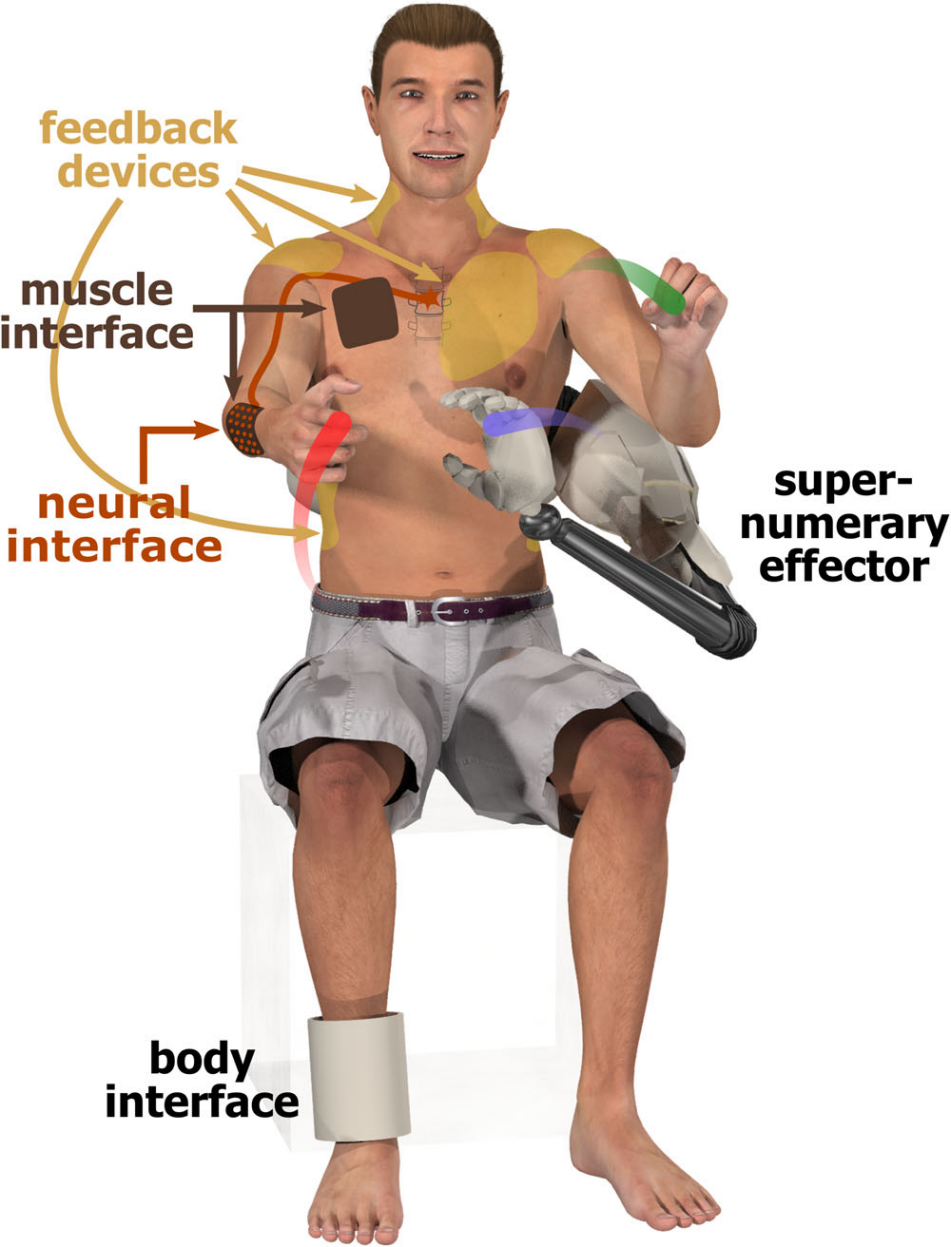
Interfaces for DoF augmentation (figure by Tobias Pistohl). An individual is augmented using a body, muscle or neural interface to control the supernumerary effector. Sensory information may be provided through feedback devices. The interfaces, supernumerary effector and feedback devices are shown at representative locations. While muscle interfaces are generally noninvasive, neural interfaces can be both invasive or noninvasive.

DoF augmentation typically includes three components (Fig. 3): The *supernumerary effector* (SE) that provides the sDoFs, the *command interface* that converts user intention into commands for the SE; and the *feedback devices*, which give the user SE status knowledge. The SE can be a robotic limb or an effector in virtual reality. It can be wearable and move with the body (Fig. 1a, d), or can be separated, e.g., a robotic arm fixed to its user’s wheelchair (Fig. 1c). In addition, the technological design can be optimized to its functional task and, thus, may vary across applications. For instance, a surgical device can be controlled by the surgeon (Fig. 1e), while a mobile phone application (Fig. 1f) may be controlled while subjects can simultaneously use their hands.

Existing SE research has mainly focused on developing supernumerary limbs, which typically comprise robotic arms^18–25^, fingers^26–31^ and legs^32–36^. Supernumerary arms are fixed to the user’s torso^20,23,24^, shoulders^18^, or elbow^22^. Applications include aircraft fuselage assembly^19^, construction^37^, and surgery^38^, while the complexity of their control has to date limited their usage. In contrast, supernumerary fingers and legs typically possess fewer sDoFs and have applications focused on aiding impaired individuals^39^ or gait support^36^. Supernumerary fingers have taken the form of either an extra thumb^27,30^ or additional stabilizing fingers^26^. Virtual SEs have been applied for studying a subject’s ability to use an SE^40–43^, or to better understand how subjects perceive augmentation through additional limbs^13,44^ or fingers^45,46^. However, to date, applications such as those in Fig. 1f have not been realized.

A command interface for SE control is required in many DoF augmentation applications. Three forms of the interface are considered (Fig. 3):

- *Body interfaces* use the measured movement or force of a body segment. Body interfaces are in general noninvasive and may use limb movement, or information coming from the head such as gaze, facial expression, or the tongue.
- *Muscle interfaces* pick up muscle activity to command the SE. Noninvasive interfaces can use surface electromyography (EMG), magnetomyography, ultrasound, or intramuscular EMG as an invasive alternative.
- *Neural interfaces* extract signals from the nervous system. Noninvasive interfaces may use electroencephalography/magnetoencephalography (EEG/MEG), functional magnetic resonance imaging (fMRI), functional near-infrared spectroscopy (fNIRS), or the spiking activity of motor units. Invasive interfaces measuring signals inside the brain, spinal cord, or muscles may also be used.

The key terminology of human movement augmentation used throughout this manuscript is listed in Table 1.

**Table 1:** Explanation of key terms used.

Term	Explanation
Human movement augmentation	Extension of a person’s natural movement abilities
Degree-of-freedom (DoF)	A direction where independent motion can occur
DoF augmentation	Movement augmentation which increases the user’s effective number of DoFs that can be controlled with some degree of independence
Supernumerary DoF (sDoF)	An extra DoF beyond the user’s natural DoFs
Supernumerary effector (SE)	An artificial virtual or physical system implementing sDoFs
Autonomous augmentation	DoF extension using autonomously controlled devices
Augmentation by transfer	DoF extension relative to a given set of tasks by re-purposing natural DoFs
Augmentation by extension	Augmentation by extending the total number of the body’s effective DoFs
Body interface	A command interface using the movement or force of a body segment
Muscle interface	A command interface that extracts control signals from muscle activity
Neural interface	A command interface that extracts control signals from the nervous system

## Autonomous augmentation

In autonomous augmentation, the sDoFs are not directly controlled by the user. Autonomous augmentation can hence be considered as a special form of human–robot collaboration with two constraints: (i) the robotic agent does not share the control of the body DoF (in contrast to robotic exoskeletons) and instead is responsible for controlling additional DoFs to those of the human user; and (ii) the augmentation device is either worn or at least operated within the same workspace and used for the purpose of augmenting the human user. Autonomous augmentation may promote precise and quick movement while minimizing additional mental effort. However, it lacks continuous knowledge of the user’s desired behavior and therefore has been mainly used in specialized applications where SEs have very constrained behaviors, such as overhead assembly^18^, aircraft manufacturing^19^, or grasping support^47^.

Autonomous augmentation’s critical problems are user intent estimation and its transformation into SE action. For simple objectives such as bracing^48^ or gait support^34^, these two problems are typically translated into control problems. In this manner, the unknown user intent is often assumed to match the defined task. For example, the intent of a system such as that in Fig. 1d may be to maintain its posture and then its action to be executed is that of a stabilizing controller^48^. Such strategies provide safe and/or optimal action, but they give no flexibility to the user, and therefore can only be applied to specialized activities.

For more complex behaviors, autonomous augmentation relies on predictive schemes requiring the current state, task knowledge, and potentially the user’s physiological measurements. Dimensionality reduction or other machine-learning algorithms^47,49,50^ have been applied to control supernumerary fingers for which the action of the limb can be well imagined, however, this approach is limited to work under a small set of desired actions. Recently, an alternative of considering the autonomous SE as the follower within a redundant leader-follower system was proposed^51^ using an observer to estimate the user’s intent. While the method does not depend on previous data, it needs a task model meaning that it is limited to known actions.

Partial autonomy, which splits the sDoF allowing for those with a well-defined function to be autonomous while other sDoF are user-controlled, represents a means to benefit from autonomous augmentation while minimizing its disadvantages^52,53^. When and how to use it is an open research topic. For example, in ref. ^53^ principal component analysis and the predictability of the natural limb motion were used to split between autonomous and directly controlled sDoFs. Nature may also show us a way to select useful automatic behaviors. In humans, reflexes complement slower voluntary actions, where for instance, long-delay reflexes compensate for dynamic coupling^54^ and contribute to controlling standing^55^. The forces exerted by a wearable SE during use are not negligible and demand the user to compensate for them^24^. When using one or more SEs in dynamic scenarios^21,24,56^, the control could implement low-level automatic compensation for dynamic coupling between the natural limbs and SEs such as to ensure the body stability, so that the user could neglect them and focus on task control.

## Augmentation by transfer

In augmentation by transfer, the human operator uses body DoFs not involved in a task to control the sDoFs and coordinate them with the natural DoFs. An example is given by excavator control, where successful excavation requires the simultaneous movement of the platform and bucket, which is achieved through simultaneous commands from the feet and hands. Compared to teleoperation that relocates DoFs, augmentation by transfer increases a task’s DoFs by redirecting DoFs not normally used in performing the intended task.

Augmentation by transfer typically uses a command input of movements^21,30,57,58^ or muscle activations^20,59^ that do not directly interfere with the task-specific motions so that the sDoFs are in the task’s null space (see the section “A taxonomy of movement augmentation”). Using volitional sDoF control with a suitable interface, augmentation by transfer enables the control of complex supernumerary limbs as has been demonstrated in enhancing dexterity^30,60^, advanced industrial settings^61^, or through the control of a 13 DoF robotic endoscope and tool system^58,62^.

A simple implementation of augmentation by transfer is to use DoFs from direct kinematic or force recordings, for instance from a 3D camera system^57^ or mechanical sliders^63^. The mental effort associated with augmentation by transfer may limit the possible movement speed and accuracy^40,42^. Pressure and bending sensors at the foot have commonly acted as an input source for seated tasks^21^ and a supernumerary thumb^30,64^. Additional sources of input have included pressure sensors in the mouth through tongue control^65^. The simultaneous use of position and force measurements demonstrates the potential of using body interfaces to control SEs with multiple DoFs^21^.

SEs can also be commanded using an actuated exoskeleton or endpoint robotic interface. Here, the interface measures position to control SEs and also provides force feedback that can facilitate control. For instance, a seated operator can use a passive foot interface placed on the ground to control four sDoF^58^. Active interfaces have been used with both feet^25^, a hand and a foot^29^, and an elbow^66^.

As augmentation by transfer requires an interface, good performance and user comfort demand that this interface fits the user’s anatomy and neuromechanics. Therefore, the interface should be adapted to the user’s characteristics such as size or movement patterns, and typical user features can inform the design^52,58^. Moreover, the mapping from user movement to SE command can be identified from an individual’s movement patterns using machine-learning techniques^58^.

Activation of muscles independent of the task can also be used, although this may result in large-signal variability. For example, EMG signals from the torso have been used to control simple supernumerary arms^20^, and the frontalis and auricularis facial muscles have been considered to control a one DoF supernumerary finger^28,67^. Despite muscles such as the Auricularis being independent of most motion, since they have an inherent function, we consider measurement of only their activation to be augmentation by transfer, since that function is impaired while they are used as an input.

sDoFs control may also be achieved using signals from the null space of the limbs used in the task^52,68^. For instance, it has been shown that the user’s arms can both generate natural motion and simultaneously control sDoFs^68^. However, such systems depend on interference between the natural DoFs and sDoFs, which has not yet been experimentally evaluated.

Some commercial surgical robots mimic augmentation by transfer, by using a clutch system to switch the control between DoFs and sDoFs^10^. While this allows a user to control sDoFs, it does not allow for simultaneous sDoF control, and was thus classified under “command augmentation”. This engineering solution, which has been found to result in inferior performance in surgical inspired tasks^62,63^, likely has a limited potential relative to augmentation by transfer.

Finally, autonomous augmentation and augmentation by transfer can also be combined using automatic motion sequences initiated by actions in the task’s null space. Here, the user provides direct commands of intent to trigger predefined sequences for controlling the sDoFs, by using signals including voice commands^22^, facial expressions^69^ or eye movements^70^. Hand gestures have also been used to trigger the movement of an artificial actuator attached to the user’s wrist^59,60^. While such systems can use more actions than is possible in typical autonomous augmentation, the control of the DoFs is still limited to activating predefined motions.

## Augmentation by extension

In augmentation by extension, the body’s DoF are extended independently from all-natural DoFs, thereby preserving the natural movement repertoire. Autonomous augmentation uses an external operator for sDOF control, while augmentation by transfer uses task-irrelevant movements. Augmentation by extension instead uses physiological signals that can be modulated without interfering with natural limb control. Studies on augmentation by extension have only recently been conducted. A fundamental question is whether humans have the neural resources to control additional DoFs without limiting other functions^71^.

Several studies have investigated the applicability of using different muscular or neural signals to control sDoFs concurrently to and independently from natural movement. Muscular activity not associated with overt movement (“muscular null space”), for example, co-contraction of muscle groups, has been used for sDoF control concurrent to isometric force generation^72^. Neural control has been considered for instance in nonhuman primates who were encouraged to perform brain–computer interfaces (BCIs) tasks by modulating the firing rates of cortical neurons simultaneously to natural limb movements^73,74^. Control could be established with motor cortical neurons that were not tuned but also with neurons that were tuned to the natural movements at similar performance as the BCI only task^74^.

In humans, 2D cursor control was achieved where one DoF was controlled by finger movements and the other by high-gamma band (70–90 Hz) electrocorticographic (ECoG) activity emerging from a motor cortical site^41^. Subjects could dissociate their ECoG signals from the originally correlated finger movements and could modulate their signals largely independently from the ongoing finger movements despite the pre-experimental association between both.

During EEG-based BCI control, human subjects could simultaneously perform overt movements^75,76^. As EEG control signals were produced by movement imagination of limbs not involved in the performed overt movements, these studies, however, follow the approach of augmentation by transfer. In a more recent study, EEG signals evoked by grasping imagination have been used to trigger pre-programmed grasping movements of a third robotic arm while subjects balanced a ball on a board with their two hands^77^. However, the sources of the EEG control signals and the simultaneity of both tasks in this study are unclear^78^.

**Fig. 4:**
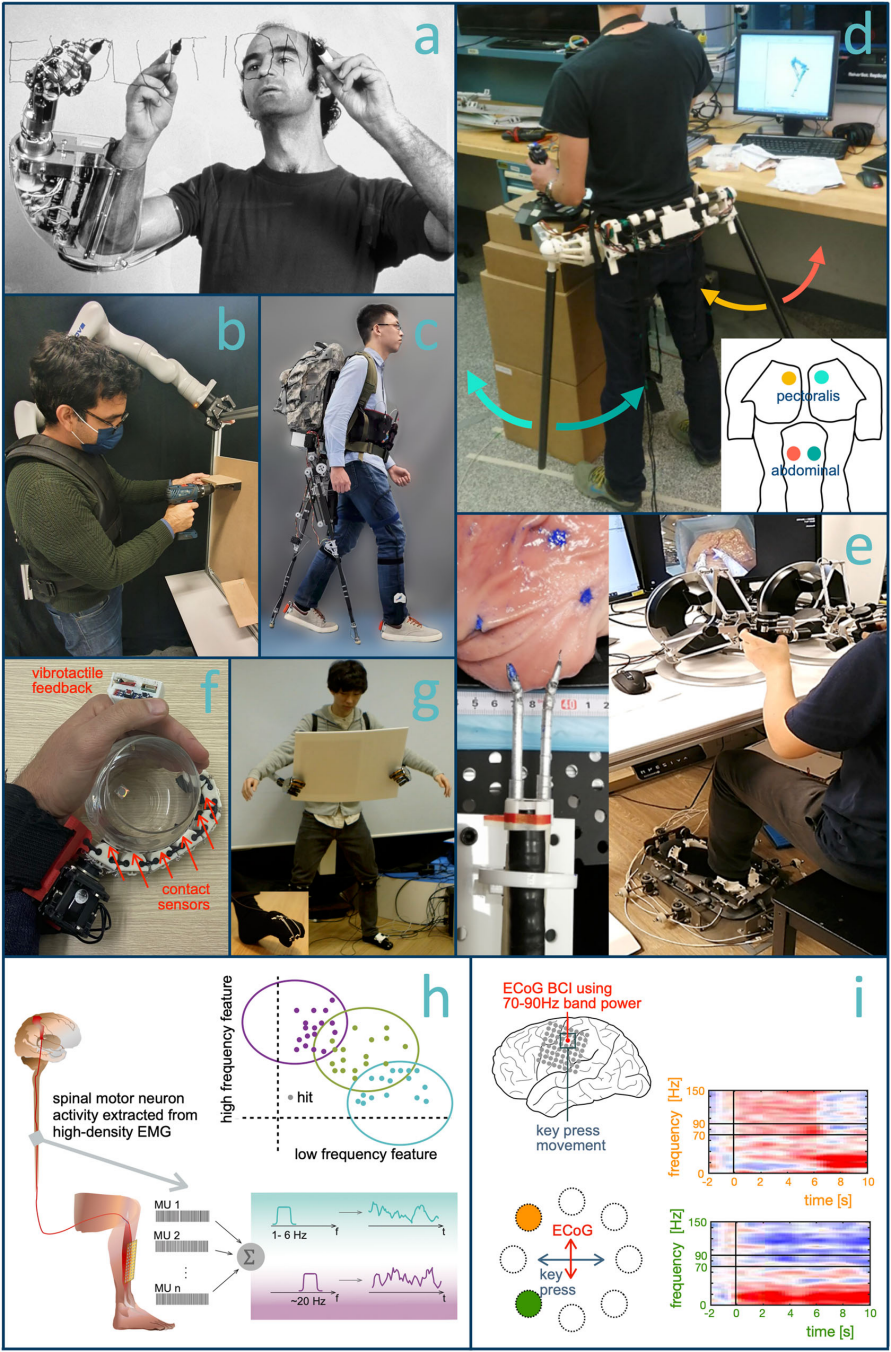
Existing systems for DoF augmentation. **a** An artistic vision of a third hand in 1982^12^. Examples of autonomous augmentation with (**b**) arm to hold a part while working on it^120^ and **c** coordinated legs to help stable walking^36^. Augmentation by transfer (**d**) with EMG control of supernumerary arms^20^ and **e** for three hand surgery with flexible endoscope^58^. Haptic feedback (**f**) for extra finger^96^ and (**g**) to control grasping with supernumerary arms commanded by the feet^21^. Towards augmentation by extension with (**h**) noninvasive interface for 2D cursor control by motor neurons from the tibialis anterior muscles^43^. Low-frequency <7Hz and 20Hz sub-band of beta activity control the horizontal and vertical cursor movements. **i** Concurrent control of high-gamma band ECoG activity and finger movement^41^ to control a 2D cursor. Time-resolved spectrograms show ECoG activity during movements to the upper and lower left targets. ECoG activity in the 70–90 Hz band, which controls movements along the vertical axis, can be modulated independently from concurrent finger movements that control movements along the horizontal axis. Panel **b** by Alexis Poignet and Mahdi Khoramshahi, **h** by Simone Tanzarella. Panel **i** modified from ref. ^41^.

For spinal motor neuron activity obtained from high-density surface EMG^79^, human subjects could partially modulate the beta band (13–30 Hz) activity without altering the force produced by the innervated muscle^43^. Moreover, subjects could control a 2D cursor using the low-frequency (<7 Hz) activity that is directly related to the force and the beta-band activity from the motor neurons of the same muscle (see Fig. 4h). Even though the beta band control remained weak, this study provides some support for using motor neuron populations in human movement augmentation. Recent results indicate that subjects could also perform 2D cursor control with three motor units from the same muscle^80^. However, subjects were instructed to perform multiple natural DoF movements which is a known way to alter motor recruitment^81^. Despite the potential existence of a neural substrate allowing for selective motor unit control^81^, a recent study, using more constrained movements, questioned whether independent recruitment of motor units could be learnt^82^.

Note that in all aforementioned studies, the overall number of controlled DoF was low, movements were simple and natural limb movement was highly restricted, not reflecting its full repertoire. Moreover, in some studies different DoFs were controlled by signals associated with different body parts, and hence the approach was effectively augmentation by transfer. Thus, no study yet has demonstrated augmentation by extension and it remains an open question whether it can be realized.

A crucial consideration for the development of augmentation by extension is the choice of the physiological control signal. Besides being independent of natural limb movement and information-rich, the accessibility of the control signal is critical as augmentation by extension aims to provide general applications. EEG and MEG offer noninvasive brain signals, however, both recordings are prone to artifacts and have limited resolution and bandwidth. Moreover, current MEG is not portable and EEG and MEG’s usability may therefore be very limited, allowing only for a small number of sDoF controlled at rather low precision and reliability. Also, fMRI and fNIRS signals may be of limited use for augmentation given their relatively low temporal resolution and in the case of fMRI their non-portability. Recordings of single units or local field potentials in the brain may offer more information-rich and movement-independent signals, yet, these signals can only be recorded invasively and may not be appropriate for many applications. An emerging technique which may allow for wearable and noninvasive control of an SE is based on the spiking activities of multiple motor units obtained from high-density surface EMG recordings^83^. Future studies will have to investigate the amount of independent control that subjects can acquire, which features of spinal motor neuron firing can be controlled, and how this can be used for movement augmentation. Besides neural signals, muscular null space signals are an interesting alternative for movement augmentation that can also be recorded in a portable and noninvasive way and should be further investigated as a candidate signal for augmentation.

## Sensory feedback

Representative existing systems illustrating the different categories of DoF augmentation are shown in Fig. 4. Current systems rely mainly on vision to control SE performance. This may limit the user’s task focus, can require significant mental effort, and is susceptible to occlusion. Studies on sensory feedback for prostheses have suggested that feedback plays a key role in enriching an artificial limb user’s experience and control^84^. Sensory feedback for sDoFs should not substitute natural limb feedback, but extend and complement it. Furthermore, this information is fundamental to achieve tentative SE embodiment, i.e., a combined internal representation together with the natural limbs which may reduce the mental effort of SE control^85^.

An often overlooked specificity of wearable SEs is that haptic feedback is intrinsically provided through the connection to the user’s body as well from the motors’ acoustic noise and vibration^52^. This may be used by the brain to model and embody the SE^86^, to acquire information on the environment^87^ or augment sensory information about the task by physical interaction with the natural limbs or collaborators^88^. In turn, this suggests that sensory feedback is more critical for a detached robot arm used as a SE or a virtual SE (Fig. 1c, e, f).

In natural limb proprioception, the sense of presence and kinematics/dynamics of body segments are known to play a central role in movement planning and execution^89^. Possible noninvasive feedback modalities to create similar artificial SE proprioception include vibrotactile motors^90,91^, electrotactile arrays^92,93^, or direct mechanical stimulation through pressure or skin stretch^94,95^. Tactile feedback has been provided for several supernumerary hands using a direct mapping of force to haptic sensation^21,96,97^. These systems have considered one or two DoF force feedback and only a few studies have considered the effect of sensory feedback to the augmentation^91,93,96^.

A number of questions need to be investigated to develop tactile and proprioceptive feedback for DoF augmentation: Would such feedback really represent added value compared to vision? Should the feedback translate position, velocity, torque or a mixture of them? Where should such feedback be relayed? And how does the feedback affect the user’s performance and SE embodiment? The answer for each of these questions will typically differ for each form of augmentation.

Augmentation by transfer uses the natural limbs not involved in a task to command a SE. Therefore, the user can rely on these limbs’ proprioception and forward model and may not need additional sensory feedback conveyed to other body parts. To enable the association of the feedback received on the natural limbs with the interaction of the SE with the environment, haptic feedback is required e.g., through a robotic interface^25,38,58^.

In autonomous augmentation, safety of operation requires that feedback be provided if or before the SE comes in contact with the body or with the environment. However, autonomous behaviors of the SE are implemented so as to discharge the SE user from controlling it in routine tasks. Therefore, continuous artificial feedback may not be required in addition to the naturally available feedback such as from vision or haptic feedback from the connection of the SE with the body. For example, the automatic compensation for dynamic coupling with the body should not be fed back as its role is to free the user from the corresponding mental effort and enable them to focus on the task.

Augmentation by extension can benefit most from a rich multimodal sensory feedback and is at the same time the most challenging class of augmentation to implement, as the motor system has no intrinsically associated sensory feedback system. Augmentation by extension allows the use of SEs in parallel to their limbs, thus noninvasive interfaces should exploit part of the body others than limbs, such as the back or the side of the trunk, the tongue, or the head. Minimally invasive interfaces could also offer a solution, such as neural interfaces implanted percutaneously^98^. For subjects with neurological impairments, other more invasive channels could be considered in the future, such as intraneural, dorsal root ganglion or epidural implants^99^. Due to their invasiveness, these solutions are likely limited in their potential application.

## Learning

The achievable performance and skill with a SE depend on its design, the control interface, and the sensory feedback it provides. However, the performance of augmentation will also critically depend on the learning carried out with the SE in order to improve task performance.

Autonomous augmentation typically requires that the user comprehends the relationship between natural limb movement and autonomous behavior. As the SE’s behavior is designed to support human action, the user should normally learn to ignore the SE’s actions and focus on their relevant subtask. However, the sDoF available may result in the user selecting new strategies to perform the task, in analogy to possible behavioral changes that drivers make in adapting to an automatic gearbox. Finally, for complex autonomous sequences, the human user may learn to predict the SE’s behavior, both for safety and coordination, similar to learning predictive models in movement interaction^88^.

Augmentation by transfer uses the natural activation of certain body segments to control the sDoFs. As humans control and coordinate their body segments from before birth, we expect that the learning requirements can be reduced by exploiting existing coordination patterns. In such cases, it is mainly the mapping of the additional limbs’ signals to the sDoF control that needs to be learned. The underlying learning process may correspond to the learning of modified visuomotor coordination^100^ and force fields^101^, which can be learned quickly^87^. Indeed, learning a simple trimanual coordination task appears to require a duration in the range of an hour^40,42^. If instead new coordination patterns, which do not belong to the subject’s natural movement repertoire, are used to control the primary DoFs and the sDoFs, extended practice may be required. The level of coordination between the primary DoFs and sDoFs achievable in augmentation by transfer will also depend on the natural coordination between the associated natural limbs. For example, the coordination between one foot and hand may not be as efficient as between the two hands^42,64^. Several works have investigated the learning of skilled actions such as the coordination of two SEs^20^ or complex manipulation with a hand equipped with a supernumerary sixth finger^28,64^. This process requires significantly more time than simple monitor tasks^40,42^.

While the learning of augmentation by extension arguably depends on the way it is implemented, it requires that subjects can learn to modulate a control signal independently from natural movement. Several studies have demonstrated a high degree of flexibility and adaptability of cortical neurons: firing rates of individual motor cortical neurons can be conditioned^102,103^ and as a result controlled independently of muscle activity^104^ and neighboring neurons^105^. New mappings from firing rates of populations of motor cortical neuron to BCI-controlled cursor movement can be learned within sessions as long as the co-modulation of neurons is maintained as in natural movement^106^. Even mappings with altered co-modulation of neurons can be learned with training spanning several days^107^. These findings demonstrate a high level of cortical neuron adaptability. They were, however, not obtained during a movement augmentation paradigm combining neural control with natural limb movements. Moreover, the reported studies were based on intracortical recordings. It remains an open question whether noninvasive recordings exhibit similar flexibility.

How does the brain adapt and reorganize during the acquisition of SE control? There is little data on this question to date, yet we expect that reorganization of the brain may occur in multiple areas, and may depend on the type of augmentation as well as on the specific sensory feedback and methodology used for augmentation. In general, augmentation by transfer may induce changes similar to those seen in learning new motor skills or learning new inter-joint and inter-limb coordination patterns. Recently, a slight shift in fMRI correlates of single digits in primary sensorimotor areas was observed when subjects learned to use a supernumerary thumb commanded by their foot^64^, as was previously observed in piano players^108^. At the same time, no changes in the hand-foot association in primary sensorimotor areas were observed in^64^ after practicing with the foot-controlled supernumerary thumb. The use of a sixth finger has also been suggested to induce changes in the corticospinal output during imagined grasping with the supernumerary finger as compared to imagined pinch and whole-hand grasping^109^. For augmentation by extension, there are currently no studies that have examined reorganization in the brain while subjects learned to use the SE. A polydactyly individual with fully functional natural supernumerary digits (six per hand) exhibited dedicated neural resources to control the extra finger, as evidenced by fMRI correlates in ref. ^4^. However, this subject was born with supernumerary limbs and therefore their brain organization is the result of genetics and a long-term developmental process. The possibly different brain reorganization in adults using SE needs to be investigated in future studies.

A possible concern about learning to use a SE is that it may deteriorate the normal natural limb control. Could learn to control a SE overload the overall repertoire of brain function? The amount of training and the level of proficiency achieved by professional athletes or musicians would suggest that the brain is able to learn an almost unlimited number of skills. However, there are reports describing how hyper-trained function can impair others, such as London taxi drivers with exceptional navigation ability at the cost of limited new spatial memory^110^. In addition to plasticity, this concern is also linked with the overall mental effort that can be handled by the brain, the ability to process information which is limited^111^.

## Application scenarios

DoF augmentation has been suggested for a range of medical, industrial and commercial applications, particularly in areas where multi-person collaboration would otherwise be required. Within laboratory settings, fields of application include aircraft fuselage assembly^48^, construction^37^, and surgery^38,62^. Wearable SEs for restorative purposes, including balance assistance^20^ and supernumerary finger’s for aiding hemiplegic stroke patients^28,59^ have also been tested on patients. However, there is currently no clear evidence to suggest that (i) subjects would have improved performance with the supernumerary limb, and (ii) the SE could be used in a practical setting given safety and regulatory constraints.

Within the different types of DoF augmentation, augmentation by transfer is likely the closest to being suitable for real-life applications, as it is based on direct user control obtained from DoFs that the user is already familiar with controlling. In particular, body interfaces such as that of^27,30,62^ exploit well-established technologies, such as pressure sensors and pedals, that users have a high familiarity with using for giving commands and therefore appear suited for potential broader trials. In contrast, autonomous augmentation is currently limited by the capabilities of intent estimation algorithms, which also adds safety/liability challenges. Augmentation by extension has yet to be demonstrated in laboratory experiments and therefore appears to be further from practical application than the other two types of DoF augmentation.

It is not yet clear which tasks are most suited for DoF augmentation due to a lack of understanding of the capability of human user’s to exploit it. In particular, the limits of the user’s ability to simultaneously coordinate their natural limbs with the SE needs to be better understood as this ability is a requirement of many of the proposed applications including robotic surgery and industrial assembly. To study the effect of the coordination between the SEs and natural limbs, we propose to identify tasks based upon (i) whether or not the dynamics of the hands are coupled (coupling); and (ii) whether or not the desired output of each hand depends on the others (dependence). Table 2 summarizes the coordination types in the case of manipulation with three limbs with examples of typical tasks. This involves typical three-handed tasks such as holding an elastic membrane, opening a door while holding a box with both hands and manoeuvring both the camera and tools in surgery. Using these coordination types, different tasks, and the relative augmentation performance may be evaluated.

**Table 2:** Coordination types of one SE and two natural limbs with representative tasks. Representative types are arranged in a matrix where the rows denote the dependence of the task goals for each hand and the columns whether there is a coupling present.

Targets hands	All coupled	Two coupled	All uncoupled
All dependent	Manipulating an elastic membrane	Camera for endoscopy	Triangulation (e.g. of laser beams)
Two dependent	Drawing on a table with the balance of the table maintained by two arms	Holding a box with two arms while opening a door with the other limb	Tying shoelaces with two hands while holding an object
All independent	Holding a board and at the same time fixing its two ends	Screwing a part to a circuit board while collecting the next part	Multi-object pick and place

Within this context, existing research has shown that without the presence of physical constraints (all uncoupled) superior performance is possible using three hands in place of two^40,63^. This is observed both when all hands are independent^40^, and when the sDoFs need to be coordinated with the natural DoF for operating a camera for position control (all dependent)^63^, as would be the case for surgery with a camera. In^40^ subjects also felt no additional mental effort for control, and in fact expressed a preference for using the sDoF in the task. When considering tasks that can only be performed with three hands, it was observed that subjects felt little change in mental effort irrespective of the condition when going from a bimanual reaching task to a trimanual task^42^. However, such coupling does result in reduced performance and when continuously tracking additionally leads to increased mental effort^42^. Finally, when comparing subject performance to that of dyads, the dyad has to date outperformed the single user controlling an SE who had the higher mental effort in all tested cases^112,113^. However, the relative difference between results appears to reduce in coupled motion (all coupled and all dependent)^112^, and may disappear when haptic feedback and some learning is provided^113^, although users still reported increased mental effort.

## Towards effective human movement augmentation

The field of DoF augmentation has exhibited a steady increase in research activity over the last decade^1,2^. In this regard, a series of pioneering studies^14,20,26,32,47^ have explored various robotic SEs and their autonomous or movement/muscle based control as well as their application to augment movement. Basic related neuroscience aspects such as natural SE^4^, multitasking, independence and coordination of SE and natural limbs^40,57^, learning^64,113^, SE embodiment^13,46^, and the feasibility of true DoF augmentation^41^, have also been investigated, providing foundational knowledge of user capability for augmentation. In this section, we analyze the limitations of current systems and propose several areas where we think future work should focus to make DoF augmentation a reality. Key open questions for DoF augmentation are also highlighted in Table 3.

**Table 3:** Key open questions for DoF augmentation.

Can DoF augmentation enhance functional abilities in real-life scenarios? Would such augmentation be deemed useful and acceptable by human users?
Is DoF augmentation by extension possible? If possible, which bodily signals can support it, and how many additional DoF is the nervous system able to control?
How does feedback for DoF augmentation alter the user’s performance and SE embodiment? How is that feedback best conveyed?
How can the brain represent supernumerary limbs? Which brain reorganization is induced by use of DoF augmentation devices? And which plasticity and computational mechanism underlie the acquisition and control of DoF augmentation?

### Autonomous augmentation

Autonomous augmentation represents a form of human–robot interaction that has been little investigated, in particular, in terms of the possible interactions and control strategies. Current systems have been restricted to simple and well-constrained applications. Partial autonomy represents one solution to expand the set of possible actions e.g., through partitioning sDoF between autonomous and manual control^53^. More complex interaction behaviors are also possible. To understand what kinds of SE behaviors could be implemented, we consider the interaction framework of^114^. A strategy that has been used to implement autonomous augmentation consists of dividing the task in independent subtasks for the human and the SE. For instance, a surgeon is in charge of the whole operation but for automatic knot tying the robot takes over. Another relatively simple autonomous augmentation strategy consists of the “assistance behavior” of^114^, where the SE control is strictly subordinate to natural limb motion. For instance, to manipulate a large object with three hands, the SE would coordinate its movements to maintain shape or a constant force with respect to the natural hands. However, more complex interaction control strategies could be used where the SE is considered as an autonomous agent. For instance, a third arm may be used for robot-assisted physical rehabilitation according to an egalitarian control scheme^115^. Rich interactive behaviors of an autonomous SE with the natural limbs may be implemented using differential game theory^114,115^.

Given that most applications of movement augmentation will require contact between a human and an artificial mechanism, the safety of that interaction must be considered before future applications are possible. While this is an issue to be considered in all forms of augmentation, its impact is likely greatest in autonomous augmentation for which the user lacks direct control of the SE. For instance, computer-controlled reflex mechanisms should be developed to prevent the SE from harming the user or a nearby person.

### Augmentation by transfer

Augmentation by transfer requires that users possess the capacity to simultaneously and independently control multiple limbs or muscles. Volitional modulation of control signals needs to be carried out together with ongoing movement. While the body can simultaneously control multiple DoFs, for instance coordinated motion of the hands and feet, movement augmentation may increase task complexity and require multitasking. The brain may have limits on the total number of DoFs it can control as well as on the complexity and number of subtasks it can carry out simultaneously. These limits will need to be experimentally investigated to determine the possible performance.

Some applications will require augmented sensory feedback, for which basic questions need to be investigated. This includes the questions of what, where and how exactly it should be? Ideally, since sensory feedback closes the loop from the user’s action to the SE’s reaction, its placement and modality should parallel the actuation and usage of the SE. The level of knowledge given by inherent feedback also needs further investigation to identify when and in which cases additional feedback modules are best suited.

Both multitasking and the exploitation of sensory feedback may improve with increased user experience. If learning to control sDoFs corresponds to skill learning, what can be achieved will also depend on the amount of training time required, e.g., months or years of training may be necessary for good performance as in sports. However, such extended training periods may not be available or desirable for all applications. Systematic research is therefore required to develop and optimize learning paradigms for the acquisition of skilled SE control.

### Augmentation by extension

The major current limiting factor in augmentation by extension lies with determining where the resources for controlling the sDoFs could come from. Two fundamental questions need to be addressed in this regard: (i) do users have sufficient independent physiological control signals? and (ii) can such signals be reliably and robustly sourced? Given the high degree of redundancy present from the neural to kinematic levels of the musculoskeletal system, we believe that in principle, the nervous system is able to learn to generate signals that can be modulated independently from movement. However, this is currently unknown, and even if the brain can generate such a signal, its reliability and dimensionality may limit the functionality of augmentation by extension.

Motor unit activities may offer a noninvasive and portable solution to provide multidimensional signals to control an SE, however it remains to be shown they can be used for augmentation. Moreover, current decomposition algorithms extracting spiking activity from surface EMG recordings in isometric conditions need to be extended to movements in order to be used for many applications.

Muscular null space signals are another noninvasive and portable alternative signal type for augmentation with initial results focusing on the use of co-contraction showing promise^52^. However, co-contraction, which could be used by both motor units and muscle signals for augmentation, is also used by the nervous system to regulate the interaction with the environment through impedance control^116^. Hence, it is important to examine whether alternative redundant muscle patterns exists that can be used to control supernumerary DoFs.

While many of these issues may be overcome in the future, the same issues on multitasking, sensory feedback, and learning as described in the previous section for augmentation by transfer need to be considered for augmentation by extension. To maximize the performance of augmented movement, future research should thus determine and document the limits of human capability and user-specific algorithms to translate physiological control signals into augmented movement. Similarly, greater understanding of learning and plasticity specifically associated with DoF augmentation is needed since most current research can only be speculatively extended to DoF augmentation. There will likely be hard limits to the complexity and number of tasks the brain can perform simultaneously, thus restricting the functional capabilities that can be achieved with augmentation by transfer and extension.

### Ethical aspects

The augmentation of human movement abilities raises important ethical questions. This includes potential concerns that movement augmentation technology may negatively affect natural motor function, change subjects’ body identity and image, reinforce inequalities or pose problems in responsibility assignment as the boundary between humans and technology becomes increasingly blurred^117^. As a result of these questions, and the potentially “unnatural” nature of the augmentation, users may reject their augmentation device as has been observed for prostheses^118^. The user’s acceptance of augmentation is an essential issue for which the determining factors have started to be studied^119^, although there are currently no established guidelines. We believe that these and further important ethical issues of movement augmentation must therefore be addressed in a multidisciplinary approach that combines neuroscience and technology with philosophical, legal, and safety aspects.

### Experiments and applications

Common to all forms of augmentation is a need for greater understanding of the underlying computational and neural mechanisms. Progress in these basic aspects of movement augmentation will require extensive experiments with human subjects to analyze: (i) the coordination and learning behaviors, (ii) the subject’s evaluation on augmentation systems (e.g., on the aspects of comfort, sense of agency, utility, and on how their use modify actions experience), (iii) restorative devices for impaired individuals that will require patient-specific protocols.

Limitations in the precision and control capabilities of the SE’s movements and in the accuracy of user’s control signal, may limit the usability of augmentation in certain applications, such as in surgery requiring precise control. These system and human limitations should be considered to design new protocols specifically for multimanual operation.

Finally, virtual SEs e.g., on computers or mobile devices are yet unexplored and may offer new and interesting possibilities for future studies as applications in augmented and virtual reality become more common.
